# GABAergic neurons differentiated from BDNF- and Dlx2-modified neural stem cells restore disrupted neural circuits in brainstem stroke

**DOI:** 10.1186/s13287-023-03378-5

**Published:** 2023-06-26

**Authors:** Xiangyue Tang, Li Wu, Juehua Zhu, Mindong Xu, Shaojun Li, Guanfeng Zeng, Shuanggen Zhu, Yongjun Jiang

**Affiliations:** 1 Department of Neurology, The Second Affiliated Hospital of Guangzhou Medical University, 250 Changgang East Road, Guangzhou, 510260 China; 2 School of Basic Medical Sciences, Institute of Neuroscience, The Second Affiliated Hospital of Guangzhou Medical University, 250 Changgang East Road, Guangzhou, 510260 China; 3 Shenzhen Longhua District Central Hospital, The Affiliated Hospital of Guangdong Medical University, 187 Guanlan West Road, Shenzhen, 518110 China; 4 Department of Neurology, People’s Hospital of Longhua, Shenzhen, 518109 China; 5 Department of Neurology, The First Affiliated Hospital of SooChow University, 899 Pinghai Road, Suzhou, 215006 Jiangsu China

**Keywords:** Brainstem, Stroke, Neural stem cells, Brain-derived neurotrophic factor, Dlx2, GABAergic

## Abstract

**Background:**

Brainstem stroke causes severe and persistent neurological impairment. Due to the limited spontaneous recovery and regeneration of the disrupted neural circuits, transplantation of exogenous neural stem cells (NSCs) was an alternative, while there were limitations for primitive NSCs.

**Methods:**

We established a mouse model of brainstem stroke by injecting endothelin in the right pons. Brain-derived neurotrophic factor (BDNF)- and distal-less homeobox 2 (Dlx2)-modified NSCs were transplanted to treat brainstem stroke. Transsynaptic viral tracking, immunostaining, magnetic resonance imaging, behavioral testing, and whole-cell patch clamp recordings were applied to probe the pathophysiology and therapeutic prospects of BDNF- and Dlx2-modified NSCs.

**Results:**

GABAergic neurons were predominantly lost after the brainstem stroke. No endogenous NSCs were generated in situ or migrated from the neurogenesis niches within the brainstem infarct region. Co-overexpressions of BDNF and Dlx2 not only promoted the survival of NSCs, but also boosted the differentiation of NSCs into GABAergic neurons. Results from transsynaptic virus tracking, immunostaining, and evidence from whole-cell patch clamping revealed the morphological and functional integration of the grafted BDNF- and Dlx2-modified NSCs-derived neurons with the host neural circuits. Neurological function was improved by transplantation of BDNF- and Dlx2-modified NSCs in brainstem stroke.

**Conclusions:**

These findings demonstrated that BDNF- and Dlx2-modified NSCs differentiated into GABAergic neurons, integrated into and reconstituted the host neural networks, and alleviated the ischemic injury. It thus provided a potential therapeutic strategy for brainstem stroke.

**Supplementary Information:**

The online version contains supplementary material available at 10.1186/s13287-023-03378-5.

## Background

The brainstem is a small part of the central nervous system that plays a crucial role in maintaining the vital signs and neurological function. A small infarct can cause the disruption of critical neural circuits, which might lead to life-threatening dysfunction [1]. Though 10% of ischemic stroke locates in the brainstem, there is a high incidence of mortality for brainstem stroke [2]. Moreover, in comparison with the anterior circulation stroke, there were more persistent and severe neurological deficits in the brainstem stroke. During the last decades, cellular therapy has been recognized as the potential treatment for numerous neurological disorders as they may reconstruct the disrupted neural circuits [3]. Based on the sources, stem cells are categorized into: embryonic, fetal, birth-related, and adult cell types [4]. Neural stem cells (NSCs) are a group of ectodermal progenitor cells that can differentiate into neurons and astrocytes [5, 6]. NSCs have been applied to treat neurological diseases [7–9], including ischemic stroke [10–13]. However, the application of NSCs to brainstem stroke remains challenging.

The post-stroke inflammatory microenvironment hinders the grafted NSCs from survival [14]. Brain-derived neurotrophic factor (BDNF), widely expressed in the nervous system, can promote the survival of neurons [15]. Our studies also showed BDNF alleviated the ischemic insult in middle cerebral artery occlusion (MCAO) model [16, 17]. Genetically modified NSCs overexpressing BDNF have been shown to improve the survival of NSCs in MCAO model [18, 19]. In addition to the survival of NSCs, there was a more crucial question: the majority of lost neurons are GABAergic neurons in the brainstem stroke [20]. Overexpression of BDNF does not promote the directional differentiation of NSCs into GABAergic neurons. Homeobox protein distal-less homeobox 2 (Dlx2), a member of the distal-less family, is a transcription factor to promote GABA synthesis, synaptogenesis, and dendritogenesis [21]. Co-overexpression of Dlx2 with achaete–scute complex homolog-like 1 in the human pluripotent stem cells could generate pure GABAergic neurons [22]. It has been hypothesized that co-overexpression of BDNF and Dlx2 promoted the survival and differentiation of NSCs into GABAergic neurons in brainstem stroke.

In the present study, we aimed to explore whether co-overexpression of Dlx2 with BDNF in the grafted NSCs could generate GABAergic neurons to restore the disrupted neural circuits in the brainstem stroke.

## Materials and methods

### Animal preparation

Adult male C57BL/6 mice (8–10 weeks, 25–35 g), provided by Animal Center of Southern Medical University (Guangzhou, China), were used in the present study. The animals were housed for at least one week before surgery under controlled environmental conditions with ambient temperature of 25 °C, relative humidity of 65%, and 12/12 h light–dark cycle. The animals were given free access to gain food and water. The animals were randomized into groups based on individual experiments to avoid the potential bias. The study design and experimental protocol were approved by the Institutional Animal Care and Use Committee of the Second Affiliated Hospital of Guangzhou Medical University (Project Title, Basic Research of Pontine infarction, No. 2019-KY-030C, Date 2019-2-18). All procedure was performed under the Guide for the Care and Use of Laboratory Animals published by the US National Institutes of Health (NIH publication No. 85-23, revised 1996).

### Brainstem stroke model

The animals were weighed and assessed for neurological manifestations prior to surgery. Induced anesthesia was performed by ketamine (100 mg/kg)/xylazine (10 mg/kg). When the animals were mounted into the stereotaxic frame, anesthetization was maintained by isoflurane (95% oxygen and 5% isoflurane, RWD Inc., Shenzhen, China) via a stereotaxic nose cone attachment for mouse (RWD Inc.) with an inlet and outlet port. After anesthesia, a 1 cm incision was made in the midline from the line of the bilateral lateral canthus to the 0.5 cm behind the posterior fontanelle. The infarction with a coordinate set of (− 5.2 mm AP, − 0.5 mm ML and − 6.5 mm DV) was performed by a 2 μl injection of ET-1 at a speed of 0.25 μl/min (ET-1, 400 μM, Sigma-Aldrich, Shanghai, China). After being in position for 10 min, the syringe was gently removed. MRI scans were used to confirm the infarct.

### MRI

MRI scanning was performed using a Bruker Biospec 7.0T system (PharmaScan, Bruker Biospin, Rheinstetten, Germany) with a mouse brain array coil and a transmit only volume coil. The anesthetized animal was secured within the cradle by tooth and ear bars, and a mouse-head 4-channel phased array surface receiver coil was placed on the head. Body temperature was maintained at 37 ± 0.5 °C during the MRI scanning procedure by a closed-circuit thermal jacket. T2-weighted scans using a fast-spin echo sequence: echo time (TE) 33 ms, repetition time (TR) 8000 ms, field of view (FOV) 30 mm × 30 mm, acquisition matrix 512 × 512, acquiring 0.4-mm-thick slices. All MR images were processed on a commercial workstation (ParaVision Acquisition 6.0.1). The infarct volume is measured by Image J (NIHSS) with a corrected standard method [23].

### Evaluation of neurological functions

In this experiment, we used the limb placement test to assess the proprioception [24]. Voetsch neuroscores were performed to assess the sensorimotor ability of vertebrobasilar system as described previously [25]. The experimental group was blind to both the two behavioral performers to avoid the potential bias.

### Isolation, culture, and differentiation of NSCs

NSCs were obtained according to a previously described procedure [26]. Briefly, the brains of E13 mice were removed and the dissected hippocampus was placed in the NSC medium containing neurobasal medium (Gibco, USA), supplemented with 20 ng/ml EGF (PeproTech, USA), 20 ng/ml bFGF (PeproTech), 2% B27 (Gibco), 1 U/ml heparin, 1% l-glutamine, 1% non-essential amino acid, 1% penicillin/streptomycin (Gibco), and 0.1 mM β-mercaptoethanol. A single-cell suspension was obtained by trypsinization with 0.05% trypsin–EGTA. Cells were grown at a density of 2 × 10^5^ cells/ml into the suitable size tissue culture task. Three days later, neurospheres emerged and transferred to a new task for the next subculture. NSCs were identified by nestin/Ki67 immunofluorescence staining.

To induce neuronal differentiation, NSCs were plated onto polylysine-coated plates at a density of 5 × 10^4^ cells/well in the NSC medium for 1 day. The NSC medium was then discarded, and the NSCs were cultured in the NSC medium in the absence of bFGF and EGF. To induce astrocyte differentiation, the NSC medium was switched to DMEM medium containing 1% fetal bovine serum (FBS). Half of the medium was exchanged every 2 days.

### Lentiviral vector production

Lentiviral shRNA vector targeting the TrkB gene was purchased from OBiO (OBiO, China). Mouse BDNF and Dlx2 genes were amplified by PCR and cloned into GL109 pSLenti-EF1-EGFP-F2A-Puro-CMV-MCS-WPRE (OBiO). Lentiviruses were constructed as previously described with slight modification [27]. Briefly, HEK293T cells were seeded at a density of 1 × 10^8^/ml and cultured in Dulbecco’s Modified Eagle Medium (DMEM, Invitrogen, USA) with 10% fetal bovine serum (FBS, Invitrogen, USA) overnight. The packaging vector was then transferred into HEK293T cells together with either the shRNA vector or the overexpressed vector or control vector. Supernatants were harvested 48 h later, followed by the determination of titer. Then, the lentiviruses were stored at − 80 °C until use.

### Transfection of lentiviral vectors

NSCs were seeded in 6-well plates coated by polylysine at 1 × 10^6^ cells/well overnight. The cells were then incubated with lentiviral vector (Lenti-BDNF, Lenti-Dlx2, Lenti-shTrkB, and Lenti-control) for 24 h. Three days later, the expressions of BDNF, Dlx2 and TrkB were analyzed by qRT-PCR.

### Transplantation of NSCs

Two microliters of NSCs (0.5 × 10^6^/μl) in PBS was injected into the mice with a coordinate set (− 5.2 mm AP, − 0.5 mm ML and − 6.5 mm DV) at a rate of 0.25 μl/min. After being in position for 10 min, the syringe was gently removed.

### qRT-PCR

Total RNA was extracted using TRIzol (Invitrogen, USA). Reverse transcription of total RNA into complementary DNA (cDNA) was carried out using the Prime Script RT reagent Kit (Takara Bio, Japan). qRT-PCR was performed using the SYBR TB Green Premix Ex Taq II (Takara Bio, Japan) in a 10 μL reaction volume on the LightCycler480 System (Roche, USA). RNA primers were purchased from Huada Gene Technology Co., Ltd. (Shenzhen, China). Primer sequences are listed in Additional file 1: Table S2. The conditions were pre-denaturation at 95 °C for 10 min, followed by 40 cycles at 95 °C for 15 s and 55 °C for 1 min followed by a dissociation stage at 95 °C for 15 s and 55 °C for 1 min. PCR reactions were repeated three times in each group, and the relative expression values were calculated using the 2^−△△CT^ method considering β-actin as internal reference control.

### HE staining

The 4 μm brain sections were mounted in the slides and stained with 2% (w/v) HE using standard methods. The slides were mounted with a non-aqueous mounting medium and a coverslip. The slides of HE were scanned by the Aperio CS2 scanner to calculate the infarct size according to the manufacture’s guideline (Leica Biosystems, San Diego, CA, USA).

### Immunofluorescence staining

The brain sections (30 μm) were mounted on positive charged plus slides, rinsed with 0.01 M phosphate-buffered saline (PBS) three times, blocked with 5% bovine serum albumin (BSA) for 2 h, and incubated with primary antibody with 0.5% Triton X-100 and 5% BSA at 4 °C overnight. The cross sections were washed three times with PBS and then incubated with the corresponding secondary antibody at 37 °C for 2 h, and DAPI was used to stain the nuclei. The sections were then over-slipped with a fluorescent mounting medium. Slides were stored at 4 °C until viewing. The sections were observed with a fluorescence microscope (Leica, Germany) or a laser confocal microscope (LSM800, Zeiss, Germany) to produce a full brain scan. The list of antibodies is shown in Additional file 2: Table S1.

Five sections were used for cell counting in each mouse. Counting was performed on six randomly chosen non-overlapping per section. Design-based stereography and systematic random sampling were employed to ensure accurate and non-redundant cell counts. Every section under analysis was a minimum distance of 100 μm from the next. The number of cells was quantized using Image-Pro software 6.0. Cell courts were performed without knowledge of experimental treatments (S. L. and G. Z.).

### TUNEL staining

The TUNEL procedure was performed according to the manufacturer’s instructions (Roche Diagnostics, catalogue # 11684817910, Shanghai, China). The 20 μm sections were incubated with blocking solution (3% H2O2 in methanol) for 10 min at room temperature, followed by washing three times with PBS. The sections were incubated for 3 min in 1% Triton X-100, followed by three washings with PBS. Then, the sections were incubated with the TUNEL reaction mixture at 37 °C for 1 h with the TUNEL reaction mixture. DAPI was applied as a nuclear counterstain. The sections were then over-slipped with a fluorescent mounting medium. Images were taken with a Leica SP8 confocal laser microscope at 400× magnification with Leica Application Suite 4.8.0 software according to the manual. Fluorescence microscopy detection: excitation wavelength 450–500 nm and emission wavelength 515–565 nm.

### Electrophysiological recording

To investigate the electrical activity of NSC-derived neurons, the whole-cell patch clamp was performed with a HEKA EPC amplifier 10 (HEKA Inc., Germany) 28 days after transplantation.

For the preparation of the brain slices, the mice were anesthetized with isoflurane. The brain was quickly removed and chilled in an ice-cold slicing liquid containing: 120 nM Choline-Cl, 2.5 nM KCl, 7 nM MgCl_2_, 0.5 nM CaCl_2_, 1.25 nM NaH_2_PO_4_, 25 nM NaHCO_3_, and 10 nM glucose. Brainstem slices (300 µm) were sectioned in ice-cold modified ACSF using a VT-1000S vibratome (Leica, Germany). Then, slices were transferred to a storage chamber with modified artificial cerebrospinal fluid (ACSF) containing: 126 nM NaCl, 3 nM KCl, 1 nM MgSO_4_, 2 nM CaCl_2_, 1.25 nM NaH_2_PO_4_, 26 nM NaHCO_3_, and 10 nM glucose. All solutions were saturated with 95%O_2_/5%CO_2_ (vol/vol). After 30 min at 32 °C followed by 1 h at room temperature (24 ± 1 °C) incubation, slices were transferred to the recording room. For the whole-cell patch clamp recording, slices were placed in the recording chamber superfused (2 ml/min) with ACSF. The GFP + cells were detected by a fluorescence lamp (pE-300, CoolLED, UK). Whole-cell patch clamp recording from GFP + cells was visualized with infrared optics using an upright microscope equipped with an infrared-sensitive CCD camera (DAGE-MTI, IR-1000E). Pipettes were pulled by a micropipette puller (P-97, Sutter instrument) with a resistance of 3–5 MΩ. Recordings were made with MultiClamp 700 B amplifier and 1550 B digitizer (Molecular Device). When recording, GFP + cells were held at − 70 mV with the pipette solution containing (in mM): 125 K-gluconate, 5 KCl, 1 MgCl_2_, 4 Mg-ATP, 0.3 Na-GTP, 0.2 EGTA, 10 HEPES, and 10 Na-phosphocreatine (pH 7.40, 285 mOsm). Membrane potential changing was evoked by injections of a series of depolarizing or hyperpolarizing pulses (from 10 pA to − 50 pA at a step of − 10 pA). Spontaneous emission was recorded in the GAP-free mode under the current clamp. Postsynaptic current (PSC) was recorded at − 70 mV holding potential under voltage clamp. Cells would be rejected if series resistance above 20 MΩ or fluctuated more than 20% of initial values. The data were filtered at 1 kHz and sampled at 10 kHz.

### Anterograde tracing of nerve tracts by AAV2/1

Two weeks after transplantation of NSCs, mice received AAV2/1 (pAAV-hSyn-mCherry-3Xflag-WPRE) in the primary motor cortex (M1 area). After anesthetization, a longitudinal incision (6–7 mm) was made at the midline above the parietal bone. To expose the motor cortex, a 1 mm^2^ hole was made in the parietal bone using a drill. The 500 nl AAV2/1 (9.24 × 10^12^ V.G./ml) was injected at a speed of 100 nl/min with the following coordinates: 1.54 mm AP, ± 2 mm ML, − 1 mm DV. The brains were harvested for cryosection 14 days after the viral injection.

### Statistics

All statistical analyses were performed using SPSS 25 (SPSS Inc., USA). Comparisons were carried out with Student’s *t* test or one-way analysis of variance (ANOVA) followed by Fisher’s least significant difference (LSD) post hoc test, as appropriate. *P* < 0.05 was considered statistically significant.

## Results

### Disrupted neural circuits in brainstem stroke

**Fig. 1:**
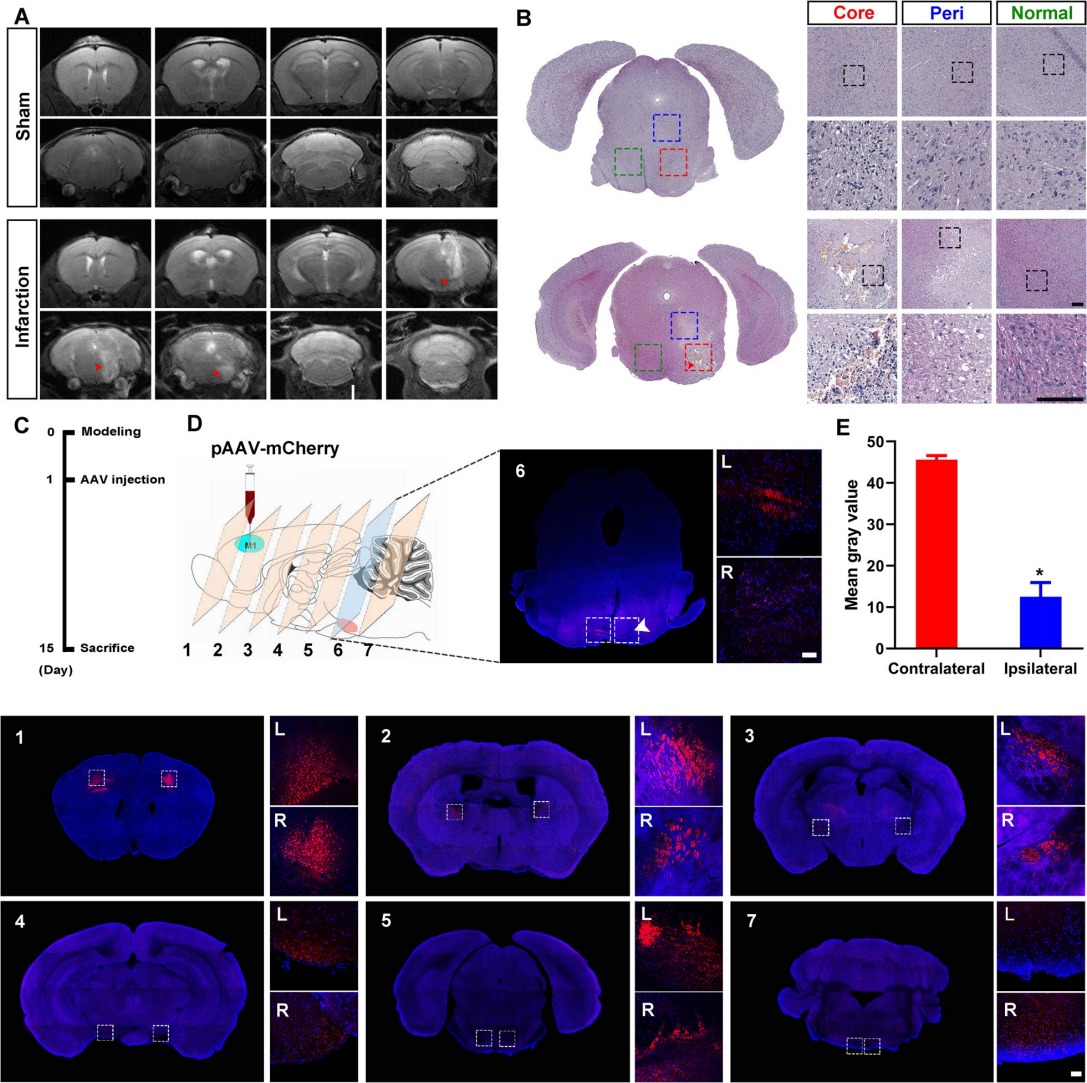
Disruptive neural circuits in brainstem stroke. The brainstem infarction was confirmed by MRI (**A**) and HE staining (**B**) 24 h after stroke onset. Infarction is marked by red arrows. **C** AAV2/1 carrying mCherry gene was injected into the primary motor cortex (M1 area) 1 day after brainstem stroke onset, and brains were harvested 15 days post-stroke. **D** Seven brain sections from the virus injection site (section 1) to the caudal portion of infarction (section 7) were made and stained with DAPI. The fluorescence intensity of mCherry within the infarct lesion (section 6) was significantly lower than that in the corresponding area on the contralateral side. Infarction is marked by white arrows. L, left; R, right. *N* = 6, scale bar = 50 μm. **E** Histogram shows the mean gray value of fluorescence intensity in section 6 measured by Image J. *P* < 0.05, versus contralateral side, *N* = 6

A mouse model of brainstem stroke was induced by injection of endothelin (ET-1) in the right pons (Fig. 1A–B). Adeno-associated virus 2/1 serotypes (AAV2/1) is an anterograde trans-monosynaptic tracer. To investigate whether the motor conduction pathway was disrupted, AAV2/1 virus carrying mCherry gene was injected into the primary motor cortex (M1 area) on Day 1 after brainstem stroke. Brains were harvested 2 weeks after AAV2/1 virus injection (Fig. 1C). A total of 7 consecutive brain sections with an interval of 1.5 mm from the virus injection site to the caudal portion of infarction were made and screened under confocal microscope (Fig. 1D). The fluorescence intensity of mCherry within or downstream the infarct lesion was significantly lower than the corresponding area on the contralateral side, suggesting the disruption of the motor conduction pathway (Fig. 1D–E).

### GABAergic neurons predominately lost in brainstem stroke

**Fig. 2:**
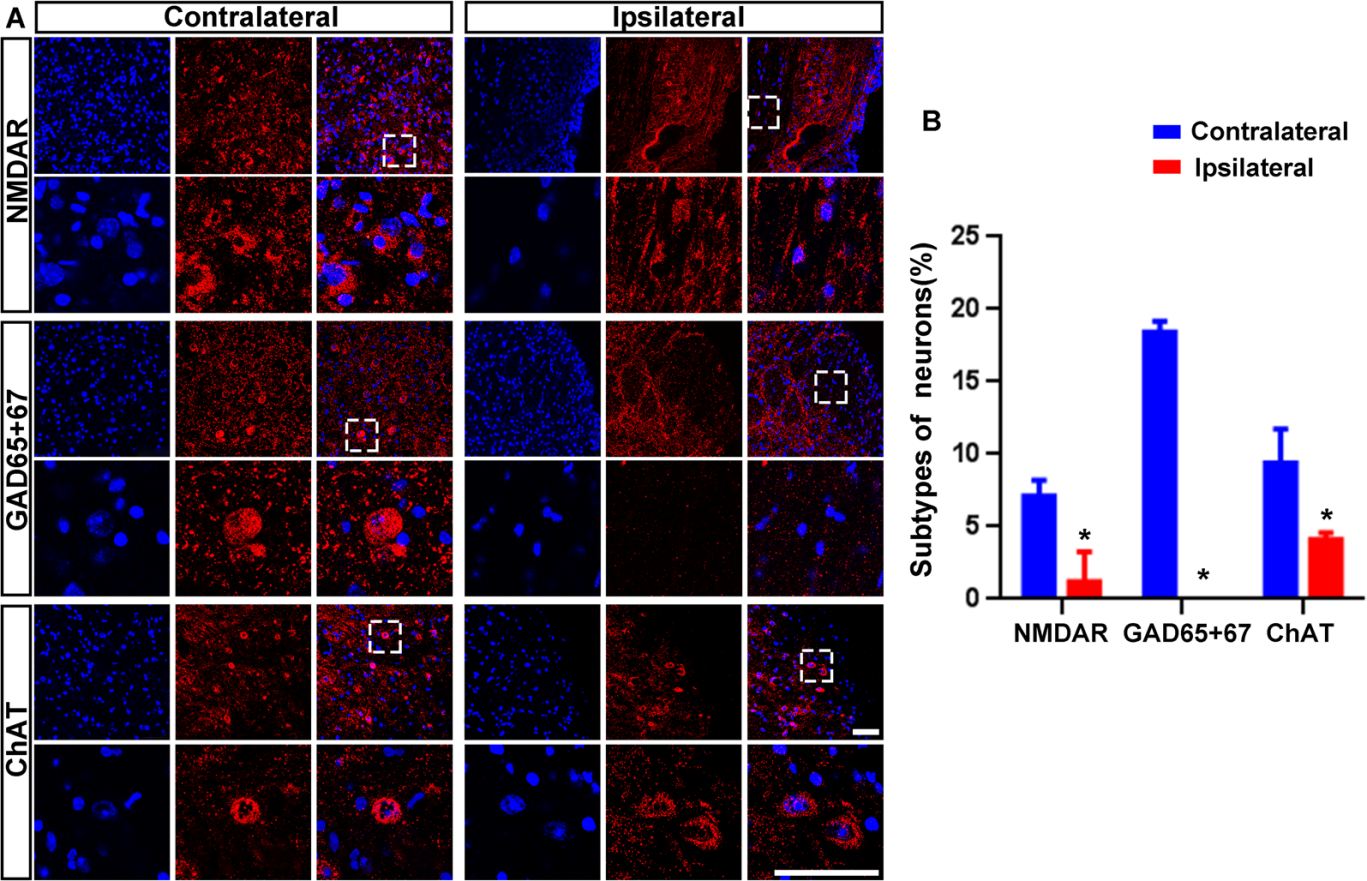
Decreased number of GABAergic neurons in brainstem stroke. **A** Brain sections were taken on Day 3 after brainstem stroke. Neurons were detected by anti-NMDAR (GLUergic) or anti-GAD65 + 67 (GABAergic). **B** Histogram shows neuron subtypes of brainstem stroke. *P < 0.05, versus contralateral. *N* = 6, scale bar = 50 μm

Diversity of neurons phenotypes has been found in different brain regions, and GABAergic neurons are the main neurons in the brainstem (Fig. 2A–B). The number of ChAT + cells, NMDAR + cells, and GAD65 + 67 + cells was significantly decreased in the infarct area when compared to contralateral side (4.09 ± 0.49% vs. 9.39 ± 2.36%, *P* = 0.019, 1.20 ± 2.07% vs. 7.10 ± 1.10%, *P* = 0.012; 0 vs. 18.42 ± 0.76%, *P* = 0.000, Fig. 2A–B), but the reduction of GAD65 + 67 + cells was much more obvious than that of NMDAR + and ChAT + cells.

### No endogenous NSCs within the brainstem infarct area

**Fig. 3:**
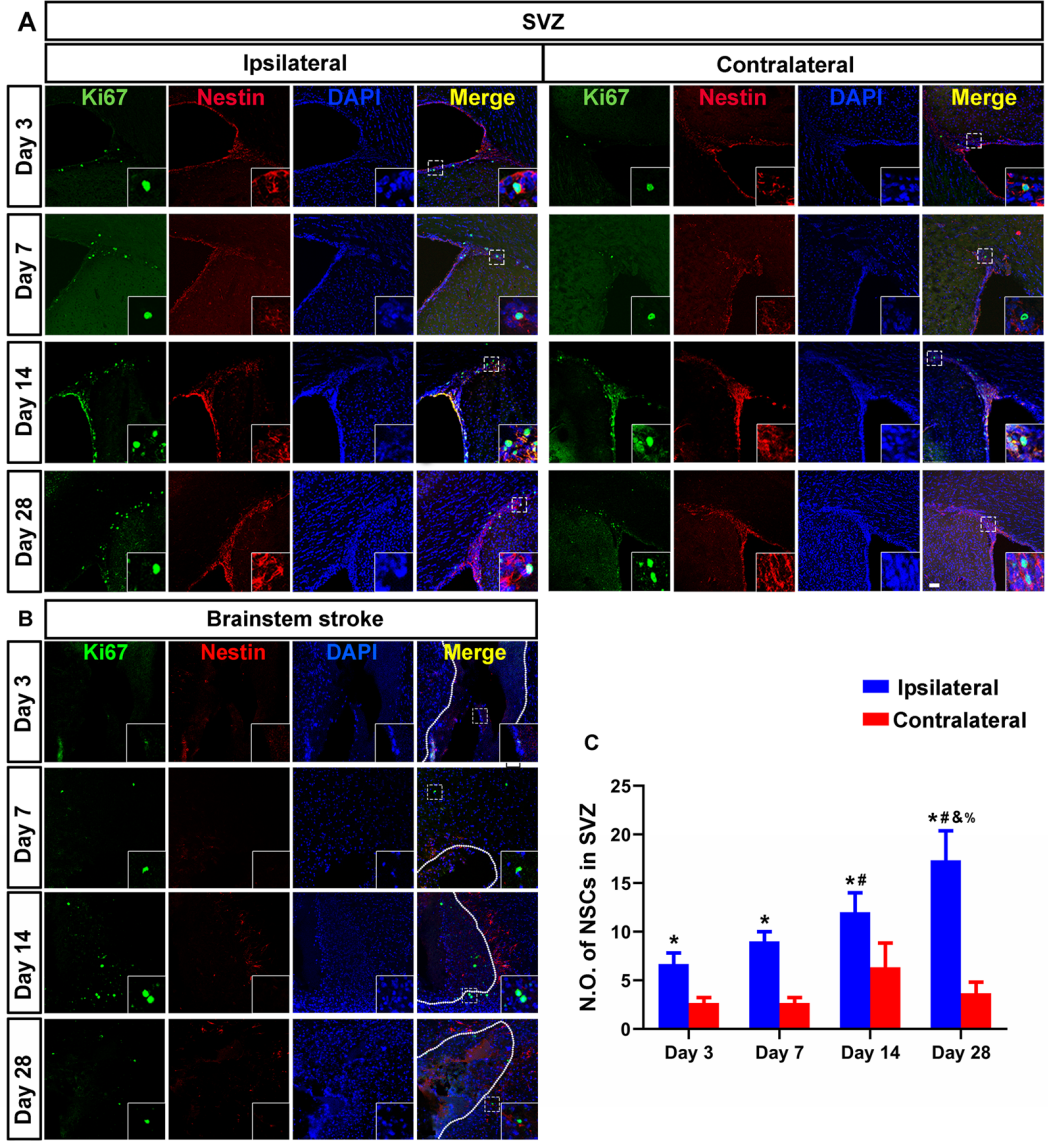
No endogenous NSCs detected within the brainstem stroke. At 3, 7, 14, and 28 days after the onset of stroke, brain sections were stained with anti-Ki67 (green), anti-nestin (red), and DAPI (blue) to detect NSCs. **A** The number of endogenous NSCs in the ipsilateral SVZ was significantly increased when compared to the contralateral side at any time point. **B** NSCs were not detected in brainstem lesions at any time point. Infarct is marked by dotted line. **C** Histogram shows the number of NSCs in the SVZ. *P < 0.05, versus contralateral side, # versus Day 3, & versus Day 7, and % versus Day 14. *N* = 6, scale bar = 50 μm

Nestin and Ki67 immunofluorescence staining were used to detect endogenous NSCs. The number of endogenous NSCs in the ipsilateral the subventricular zone (SVZ) was increased when compared to the contralateral side since Day 3 after stroke and continued to increase over time (Fig. 3A–C). On Day 28, endogenous NSCs were also found but not increased in the subgranular zone (SGZ) (Additional file 3: Fig. S1). However, there were no NSCs detected around the brainstem infarcted area at all time points (Fig. 3B). These results showed that no endogenous NSCs were generated in situ or migrated from neurogenesis niches (SVZ or SGZ) within the brainstem infarct area.

### Optimal time of grafted NSCs after brainstem stroke

**Fig. 4:**
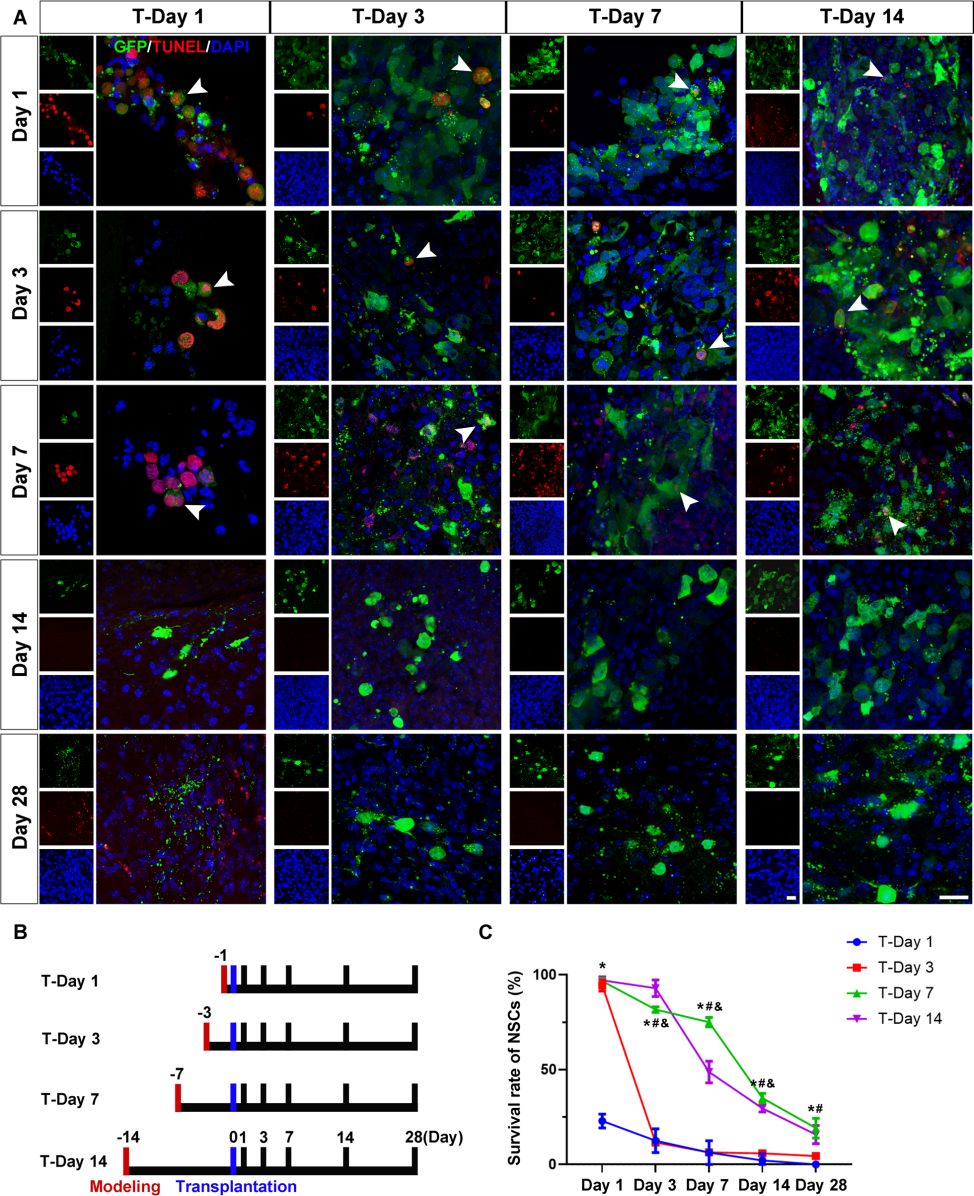
Low survival rate of grafted NSCs in the brainstem stroke model. **A** GFP-NSCs (green) were transplanted 1 day (T-Day 1 group), 3 days (T-Day 3 group), 7 days (T-Day 7 group), and 14 days (T-Day 14 group) after stroke onset. Brains were harvested on Day 1, 3, 7, 14, and 28 after transplantation. TUNEL staining (red) was used to detect apoptotic cells. **B** The schematic diagram of time points and groups. **C** The line chart showed the survival rate of the transplanted GFP-NSCs. Survival rate = $\frac{{{\text{No}}.\;{\text{of}}\;{\text{GFP}} + {\text{cells}}\;{\text{on}}\;{\text{Day}}1 - {\text{No}}.\;{\text{of}}\;{\text{TUNEL}} + {\text{GFP}} + {\text{cells}}}}{{{\text{No}}.\;{\text{of}}\;{\text{GFP}} + {\text{cells}}\;{\text{on}}\;{\text{Day}}1}} \times 100$. *P < 0.05, versus T-Day 1, #*P* < 0.05, versus T-Day 3, &*P* < 0.05, versus T-Day 14. *N* = 6, scale bar = 20 μm

In the absence of endogenous NSCs in the infarct region, transplantation of exogenous NSCs would be an alternative treatment for brainstem stroke. To track the cell fate of the grafted NSCs in vivo, we transfected the NSCs with a lentiviral vector carrying the EGFP gene. GFP-NSCs were transplanted into the brainstem 1 day (marked as T-Day 1 group), 3 days (T-Day 3), 7 days (T-Day 7), or 14 days (T-Day 14) after stroke (Fig. 4B). In the T-Day 1 group, almost no live grafting NSCs were found on Day 28. There were 0%, 4.45 ± 0.48%, 19.17 ± 5.20%, and 15.75 ± 4.82% grafted NSCs survived in T-Day 1, T-Day 3, T-Day 7, and T-Day 14 groups on Day 28, respectively (Fig. 4A–C). These results indicated that the optimal time for NSC transplantation was Day 7 post-stroke.

### BDNF promoted the differentiation of grafted NSCs into neurons

**Fig. 5:**
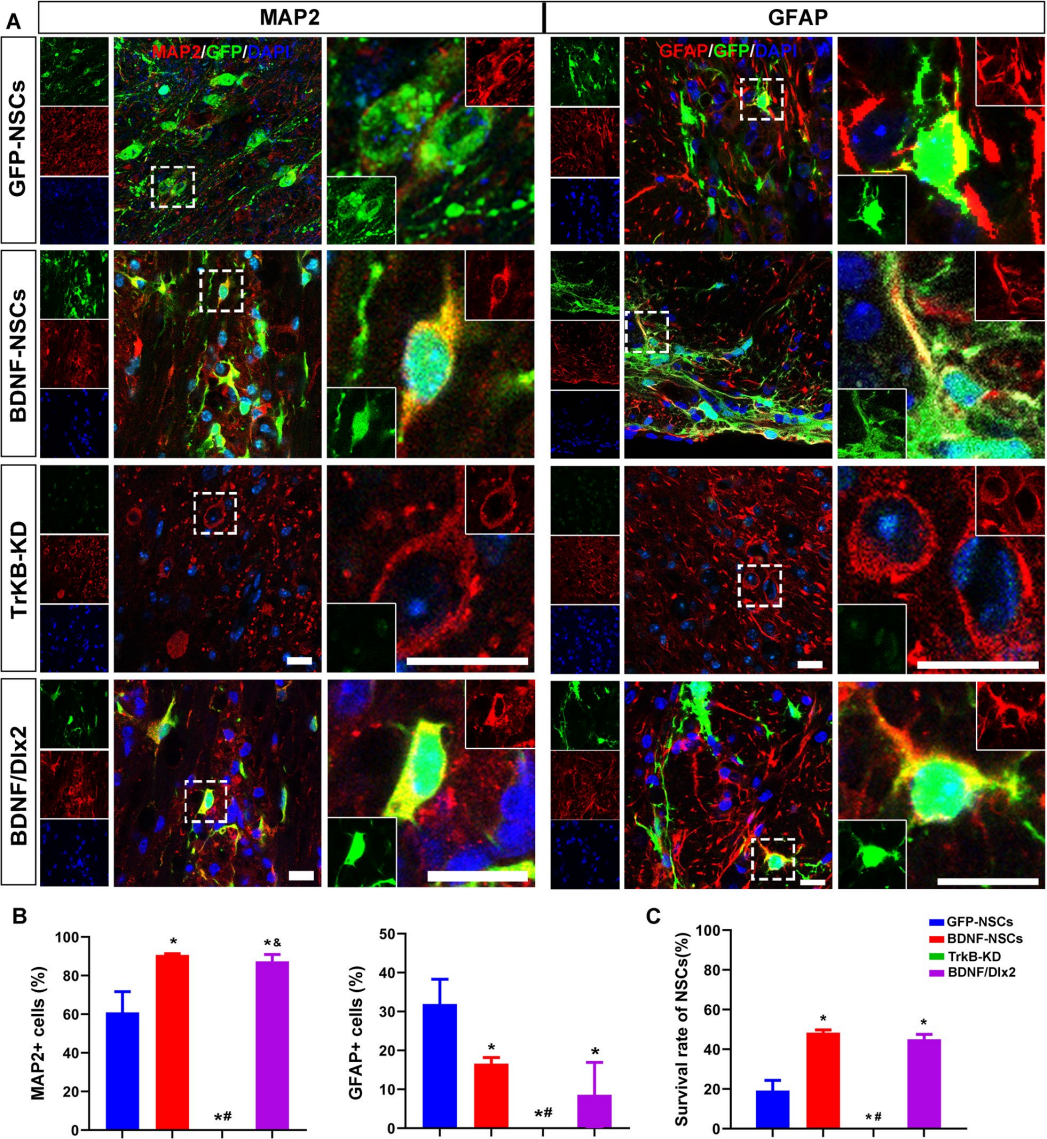
BDNF promoted the survival and differentiation of NSCs. The NSCs in the GFP-NSCs, BDNF-NSCs, and TrkB-KD groups were transfected with Lenti-EGFP, Lenti-BDNF, and Lenti-shTrkB, respectively. The NSCs were transplanted on Day 7 after the stroke, and the brains were harvested on Day 28 after transplantation. **A** Neurons were detected by anti-MAP2 (red). Astrocytes were detected by anti-GFAP (red). **B** Histogram shows the percentages of MAP2 + neurons and GFAP + astrocytes. **C** Histogram shows the survival rates of NSCs. *P < 0.05, versus GFP-NSCs, #*P* < 0.05, versus BDNF-NSCs. *N* = 6, scale bar = 15 μm

**Fig. 6:**
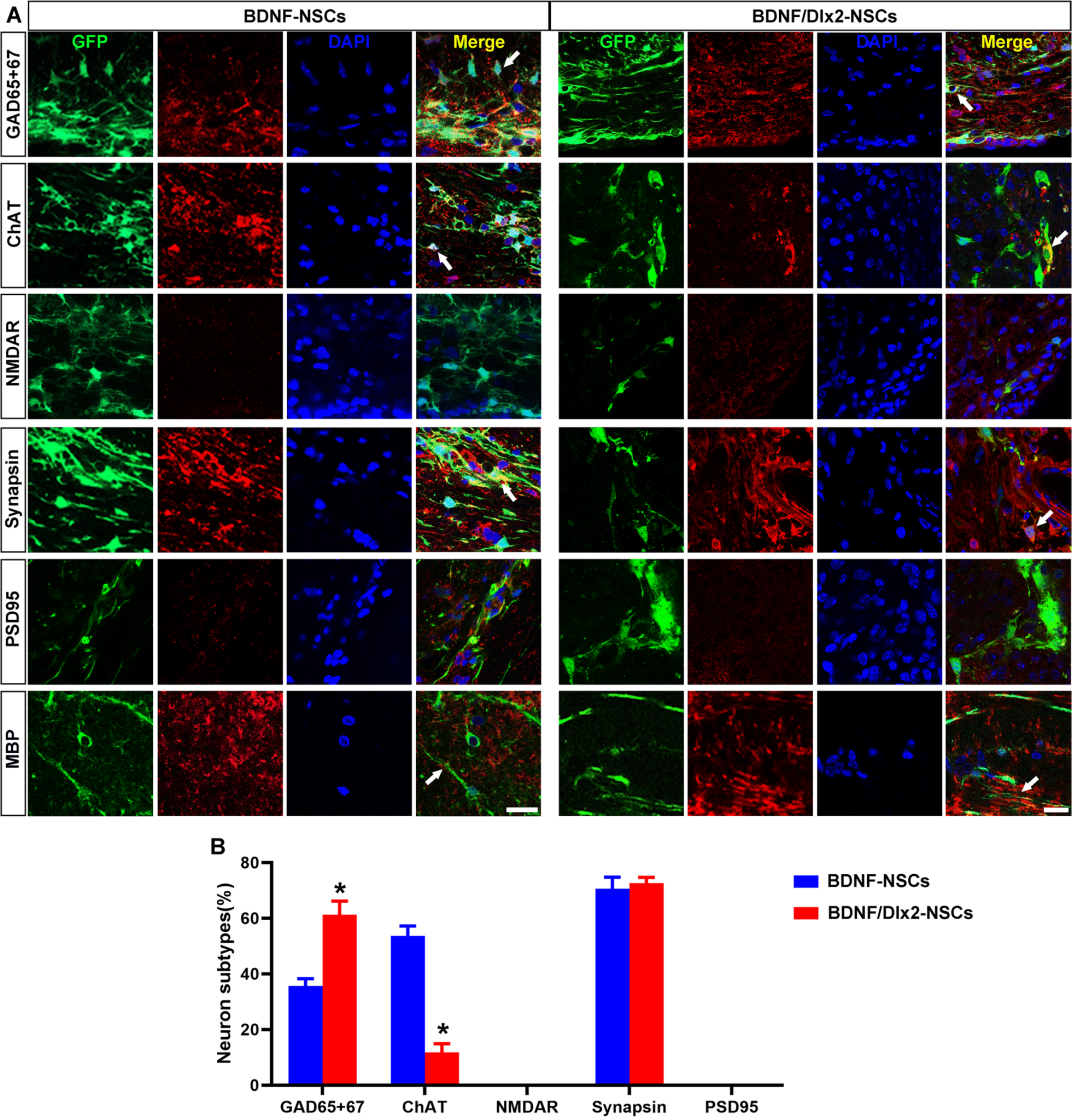
Overexpression of BDNF and Dlx2 promoted NSCs into GABAergic neurons. **A** On Day 28 after transplantation, brain sections from BDNF-NSCs (transfected with Lenti-BDNF) and BDNF/Dlx2-NSCs (transfected with Lenti-BDNF/Dlx2) groups were used to detect the phenotypes of GABAergic neurons, glutamatergic neurons, and cholinergic neurons differentiated from NSCs, using anti-GAD65 + 67, anti-NMDAR, and anti-ChAT. GFP + cells expressed synapsin, but PSD95, an excitatory postsynaptic membrane marker, was less expressed. MBP, a marker of myelin sheath, was found around GFP + cells. **B** Histograms showed the percentages of neurotransmitter markers GAD65 + 67, ChAT, NMDAR-positive cells and of synaptic markers synapsin and PSD 95 in GFP + cells. *P < 0.05, versus the BDNF-NSCs group. *N* = 6, scale bar = 20 μm

Transplanted NSC has the potency to differentiate into neuronal cells, and we further demonstrated whether BNDF modification helped NSCs to be transplanted into neurons. The mRNA level of BDNF was significantly increased in the NSCs transfected with Lenti-BDNF when compared to that with control vector (Additional file 4: Fig. S2A). NSCs transfected with Lenti-BDNF were transplanted around the site of the infarct on Day 7 after the stroke. The survival and differentiation of neurons and astrocytes from the grafted NSCs were examined on Day 28 after transplantation. On Day 28 after transplantation of NSCs, the survival rate in the BDNF-NSCs group was 48.33 ± 1.44%, significantly higher than that in the GFP-NSCs group (Fig. 5A–C). As shown in Fig. 5, compared to the GFP-NSCs group, the number of MAP2 + cells in the BDNF-NSCs group was significantly higher (90.73 ± 0.67% vs. 60.99 ± 10.73%, *P* = 0.009), while the GFAP + cells was much lower (16.62 ± 1.59% vs. 31.94 ± 6.36%, *P* = 0.016). However, most of grafted BDNF-NSCs-derived neurons were ChAT + cells, while only 35.69% were GAD65 + 67 + cells (Fig. 6A–B).

To further verify the effect of BDNF on NSCs, the expression of TrkB, a receptor for BDNF, was knocked down by Lenti-shRNA. The mRNA level of TrkB was decreased by 76% (Additional file 4: Fig. S2A). On Day 28 post-transplantation, the grafted NSCs in the TrkB-KD group were scarcely visible (Fig. 5A–C).

### Co-expression of Dlx2 with BDNF promoted the directional differentiation of grafted NSCs into GABAergic neurons

Dlx2 has been reported to promote the differentiation into GABAergic neurons from human pluripotent stem cells [21]. As suggested above, GABAergic neurons were predominantly lost in the brainstem after ischemic injury. To explore whether Dlx2 facilitated the differentiation of NSCs to GABAergic neurons, Dlx2 was co-expressed with BDNF via lentiviral vector, Lenti-BDNF/Dlx2 (Additional file 4: Fig. S2B). In the BDNF/Dlx2-NSCs group, 11.79 ± 3.11% of grafted neurons were ChAT + cells, and 61.27 ± 4.89% were GAD65 + 67 + cells, indicating that the majority of grafted NSCs differentiated to GABAergic neurons. Remarkably, these graft-derived neurons formed synaptic structures (70.56 ± 4.19% for BDNF-NSCs and 72.61 ± 2.07% for BDNF/Dlx2-NSCs, PSD95-, and synapsin + , Fig. 6A–B).

### Grafted NSCs-derived neurons integrated with host neural circuits

**Fig. 7:**
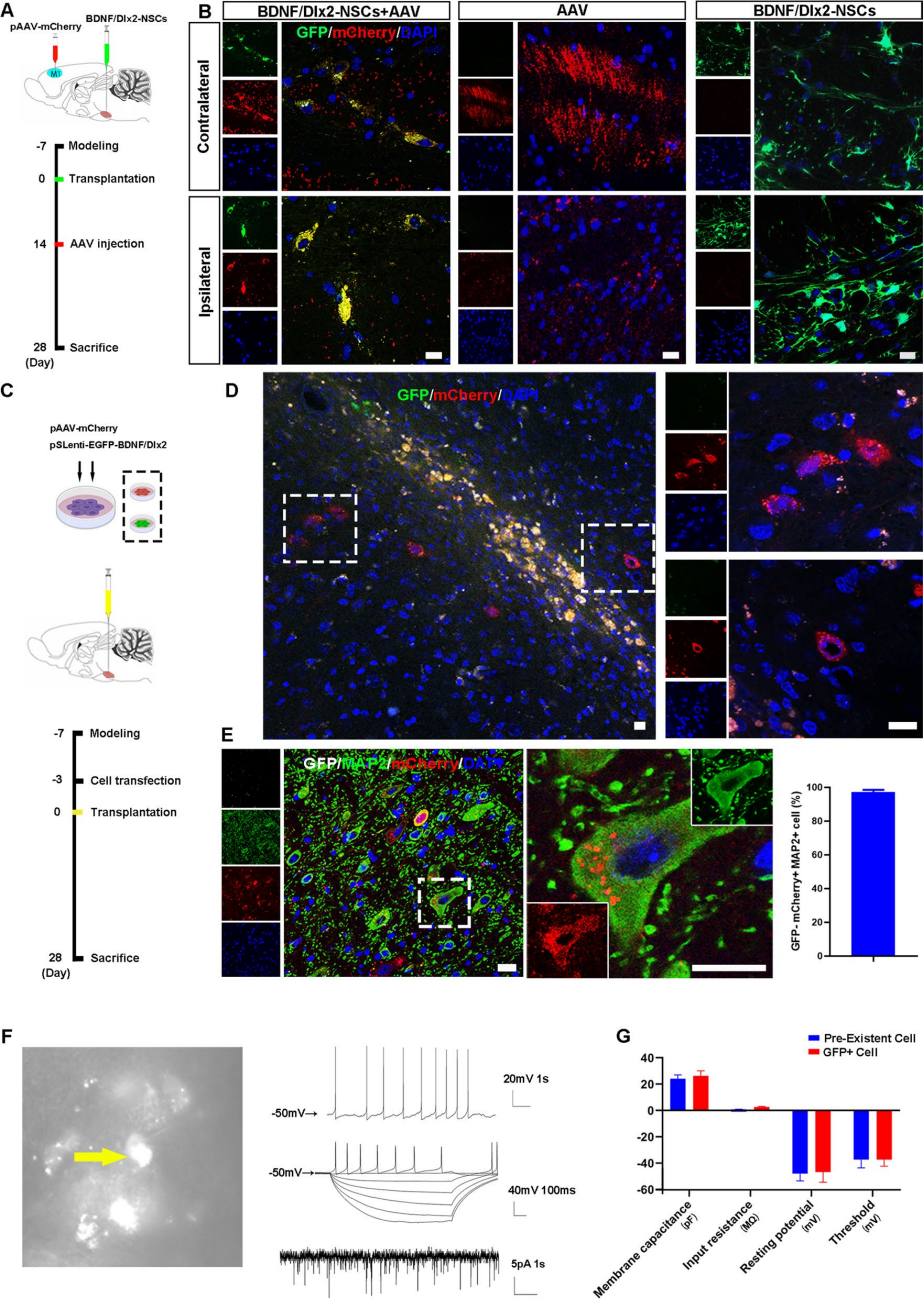
Grafted NSCs-derived neurons integrated with the host neural circuit. **A** NSCs transfected with Lenti-BDNF/Dlx2 were transplanted 7 days after brainstem stroke, and 14 days after the transplantation, anterograde trans-monosynaptic tracer AAV2/1-mCherry was injected into the bilateral primary motor cortex (M1), and brain was taken on Day 28. **B** GFP + mCherry + cells were observed on the contralateral side and ipsilateral side of infarction in the AAV-BDNF/Dlx2-NSCs group (left), but not in the control groups-AAV group (middle) and BDNF/Dlx2-NSCs group (right). *N* = 6, scale bar = 20 μm. **C** The schematic diagram of experiment design. **D** mCherry + GFP- cells represented host cells receiving substances from grafted NSCs. **E** GFP- mCherry + MAP2 + cells represented host neurons receiving substances from grafted NSCs. *N* = 6 refers to 6 mice, and each mice had 6 patched cells, scale bar = 20 μm. **F** GFP + cells (yellow arrow) were recorded by whole-cell patch clamp on Day 28 after transplantation. GFP + cells had spontaneous action potentials (upper right) and fire action potentials (middle right) and displayed spontaneous postsynaptic currents (bottom right). **G** The electrophysiological parameters of membrane capacitance, input resistance, resting potential, and threshold of GFP + cells were similar to those of host neurons. *N* = 6 refers to 6 mice, and each mice had 6 patched cells, *P < 0.05

The morphological integration of graft-derived neurons with host neural circuits was determined by anterograde trans-monosynaptic tracer AAV2/1 expressing red fluorescent protein (mCherry). On Day 14 after the transplantation of NSCs, AAV2/1 was injected into the bilateral primary motor cortex (M1), and brain was taken on Day 28 (Fig. 7A). GFP + mCherry + cells were observed in the brainstem, suggesting that the grafted NSCs-derived neurons had synaptic connections with the host neurons and received transsynaptic substances. The AAV group and the BDNF/Dlx2-NSCs group, which only received pAAV2/1-mCherry injection or BDNF/Dlx2-NSCs transplantation, respectively, showed no GFP + mCherry + cells (Fig. 7B).

Next, we co-transfected the NSCs with Lenti-BDNF/Dlx2 and AAV2/1-mCherry viruses to investigate whether the graft-derived neurons were able to send synaptic substances to the host neurons in an anterograde manner. The co-transfected NSCs were transplanted in the brainstem on Day 7 after stroke onset (Fig. 7C). On Day 28, the brains were harvested. GFP-mCherry + cells were detected (Fig. 7D). To further investigate the cell types of GFP-mCherry + , brain sections were stained with MAP2. Interestingly, almost all the GFP- mCherry + cells were MAP2 + (Fig. 7E), which suggested that these cells were host neurons and received AAV2/1-mCherry from grafted NSCs. These results showed that the grafted NSCs-derived neurons were able to receive and send synaptic substances to establish a connection with the host neuron.

To investigate whether the graft-derived neurons were functionally integrated with the host neural circuits, we performed whole-cell patch clamp recordings on Day 28 post-transplant. The results showed that the graft-derived neurons had spontaneous action potentials, fire action potentials in response to synaptic activation, and spontaneous postsynaptic currents, indicating that these neurons were electrically active and capable of firing action potentials and of receiving synaptic inputs (Fig. 7F–G). In addition, graft-derived neurons had similar electrophysiological properties with the host neurons (Fig. 7G).

### Grafted NSCs reduced infarct volume and improved neurological function after brainstem stroke

**Fig. 8:**
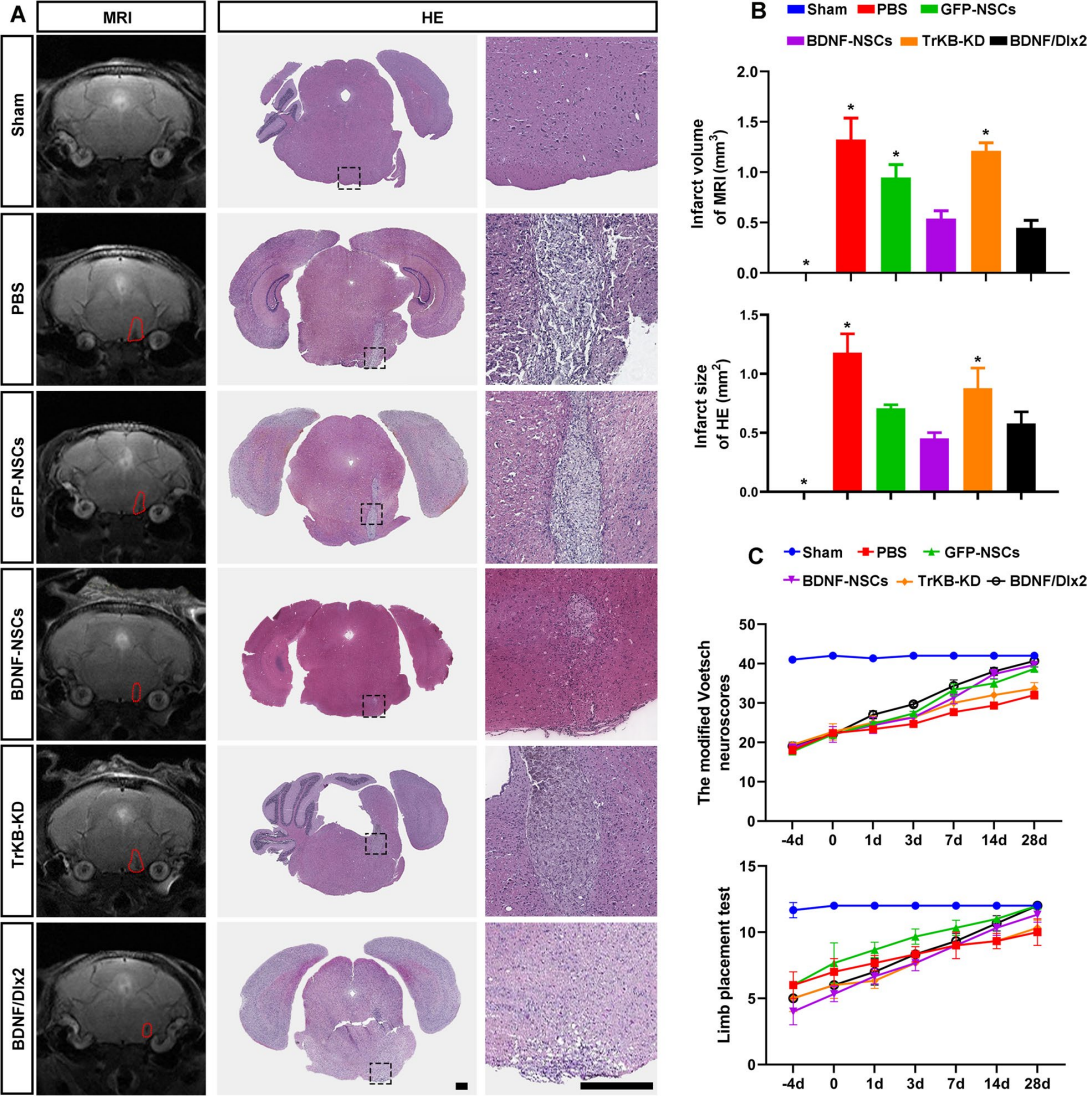
NSCs transplantation treated brainstem stroke. **A** MRI and HE staining were performed to measure and confirm the infarct volume. *N* = 6, scale bar = 300 μm. **B** The infarct volume measured by MRI and infarct size confirmed by HE staining on Day 28 after transplantation were significantly smaller in the BDNF/Dlx2 group when compared to the PBS group, GFP-NSCs group, BDNF-NSCs group, and TrKB-KD group. N = 6, *P < 0.05, versus the BDNF/Dlx2 group. (C) On Day-4, 0, Day 1, Day 3, Day 7, Day 14, and Day 28 after transplantation, the Voetsch score (above) and limb placement test (bottom) were applied to evaluate the neurological function, and the line charts showed the behavioral test scores in Sham group, PBS group, GFP-NSCs group, BDNF-NSCs group, BDNF/Dlx2 group, and TrKB-KD group. *N* = 6, *P* < 0.05

As shown in Fig. 8A–B, the MRI determined infarct volume decreased after NSCs transplantation, which was more significant in the BDNF/Dlx2 group. HE staining confirmed the MRI findings. After four days of training, on Day 0, Day 1, Day 3, Day 7, Day 14, and Day 28, Voetsch scores and limb placement tests were applied to assess the neurological function. The Voetsch score was lower in the PBS and TrkB-KD groups than that in the BDNF/Dlx2 group (Fig. 8C). The limb placement test showed similar results (Fig. 8C).

## Discussion

**Fig. 9:**
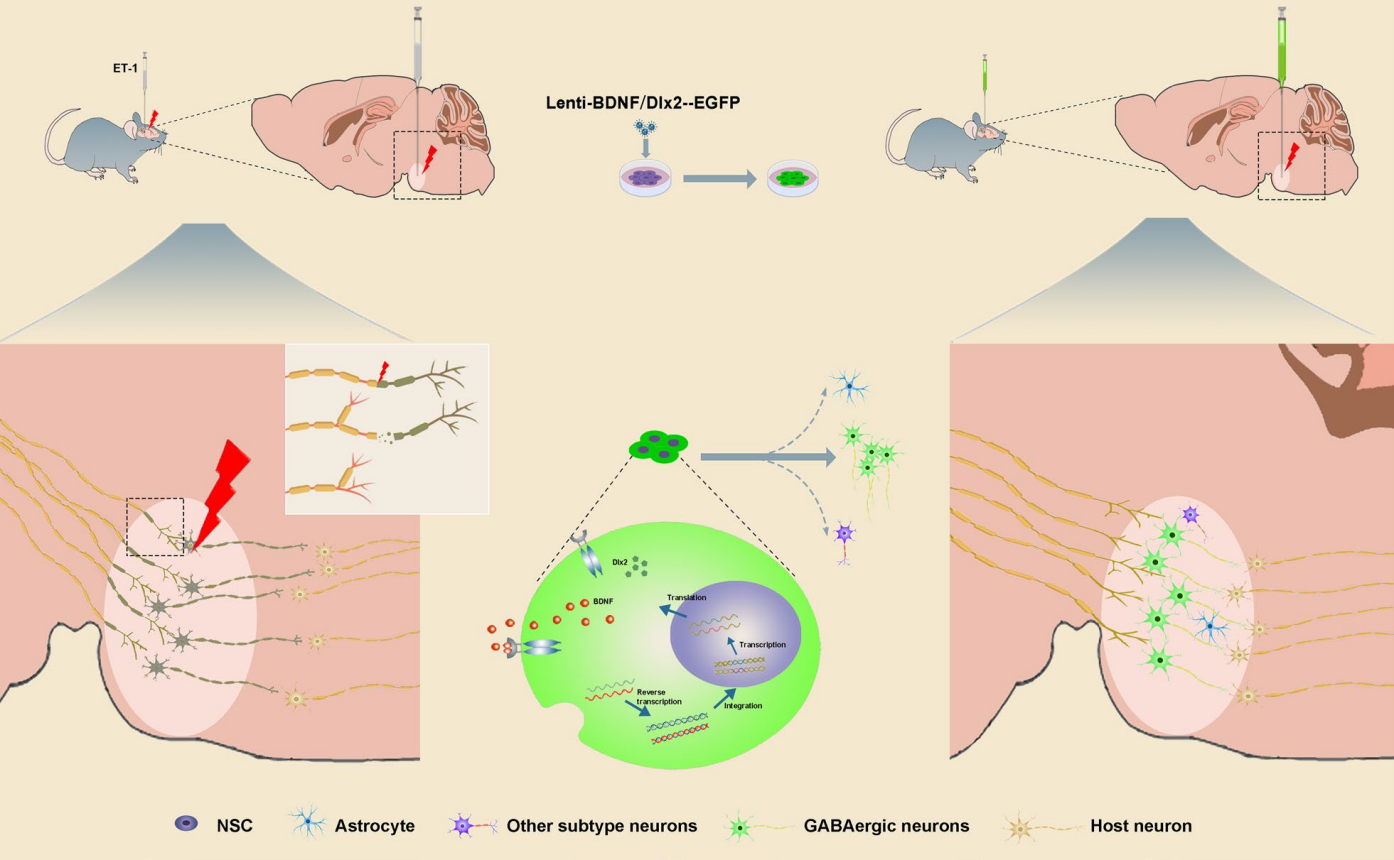
Diagrammatic summary. After transplantation of Lenti-BDNF/Dlx2-NSCs into a brainstem infarct lesion, overexpression of BDNF improves the survival of NSCs and promotes differentiation of NSCs into neurons, presumably by autocrine action, and the overexpression of Dlx2 boosts the differentiation of NSCs into GABAergic neurons. Graft-derived GABAergic neurons establish synaptic connections with host neurons and reconstruct neural circuits

In the present study, there are four main findings. First, there were no in situ or migrated endogenous NSCs engaged in restoring disrupted neural circuits in the brainstem stroke. Second, due to the low survival rate and the low rate of differentiation rate to neurons, transplantation of primitive NSCs had limited therapeutic effect. Third, co-overexpression of BDNF and Dlx2 promoted the directional differentiation of the grafted NSCs into GABAergic neurons, the dominant subtype of neurons lost in brainstem infarction. Finally, grafted NSCs-derived GABAergic neurons formed functional synaptic connections with host neurons, improved motor and sensory recovery and reduced infarct volume (Fig. 9).

The brainstem, just a half-inch in diameter, is the center for activities such as consciousness, motor and sensory control. A small infarct can cause severe neurological impairment, as demonstrated in the present study. Due to the disrupted nerve tracts, the motor and sensory dysfunction persisted for at least 28 days after the stroke. Previous studies have shown the therapeutic effect of endogenous NSCs on MCAO model [28]. However, no in situ or migrated endogenous NSCs were found in or around the infarct in the brainstem stroke. Therefore, transplantation of exogenous NSCs seemed to be the optimal option for brainstem stroke.

The primary problem for exogenous NSCs is the low survival rate because of the harsh post-stroke microenvironment caused by activated inflammatory cascades and accumulation of reactive oxygen species [29]. We found that almost no exogenous NSCs survived unit Day 28 after stroke if they were transplanted 1 or 3 days after stroke onset. Therefore, establishing an optimal time point for transplantation should take into account both the host microenvironment and the need for early rescue and replacement of ischemic and hypoxic neurons. Several time points (Day 1, 3, 7, and 14 after stroke) were tested for transplantation, and found the survival rate was highest if NSCs were transplanted on Day 7. Postponing the transplant until Day 14 or 28 did not further improve the survival rate further or even decrease it. Thus, the option time for the transplantation was Day 7 after brainstem stroke.

Even when the transplant timing was optimized, the survival rate of NSCs was still unsatisfactory for treatment. A high dose of NSCs might be a simple and easy way to do this, but it may also exacerbate cell death due to a relative shortage of oxygen and energy. Many methods have been applied to improve the live capability of NSCs [30, 31], modifying NSCs to overexpress neurotrophic factors, such as neurotrophin-3 [32] and BDNF [18]. BDNF and its receptors are widely expressed in the nervous system and can protect existing neurons, induce neurogenesis, and promote synaptic plasticity [33]. In this study, NSCs were transfected with lentiviral vectors to overexpress BDNF during the pre-transplantation phase. Overexpressed BDNF protected the NSCs against ischemic insult in vitro and in vivo. In addition, NSCs could be induced to differentiate into astrocytes and neurons in vitro. Overexpressed BDNF promoted NSCs to differentiate into mature neurons. Knockdown of the BDNF receptor, TrkB, abolished these effects.

Overexpressed BDNF promoted differentiation of the grafted NSCs into cholinergic neurons and GABAergic neurons. A brainstem stroke, however, resulted mainly in the loss of GABAergic neurons. How to make directional differentiation of NSCs into the GABAergic neurons remained undermined. In the present study, co-overexpression of Dlx2, which is a marker gene for GABAergic neurons, forced the differentiation of the grafted NSCs into GABAergic neurons. It was supported by previous studies that lentiviral vectors carrying Dlx2 gene induced pluripotent stem cells or other cells in situ into GABAergic neurons [34].

Morphological differentiation and maturation does not equate to neural network repair and functional recovery, and it remained to be verified whether graft-derived neurons integrate with host neurons. On Day 28 after BDNF/Dlx2-NSCs transplantation, graft-derived neurons were detected to have long protrusions, most extending laterally to the sides of the infarct region, with a small fraction extending longitudinally to the rostral and caudal regions. Most of the graft-derived neurons formed mature synaptic structures with the host neurons. Furthermore, an anterograde monosynaptic virus, AAV2/1, carrying the mCherry gene was injected into the primary motor cortex to investigate whether the substance could cross the synaptic structure from the host neuron to the grafted NSCs-derived neuron. Red fluorescence was found in the grafted NSCs-derived neurons. In addition, the AAV2/1-mCherry was used to transfect the NSCs, which were subsequently implanted in the infarct region. On Day 28, mCherry fluorescent protein was found in a wide range of host neurons, suggesting that AAV2/1 was anterogradely transmitted to host neurons from graft-derived neurons via newly established synapses. All these data suggested that the graft-derived neuron received synaptic transmission from the host neuron and sent a transsynaptic signal to the host neuron. Finally, electrophysiological analysis was applied to confirm the functional connectivity of the graft-derived neurons to the host neural circuit. On Day 28 after transplantation, whole-cell patch clamp recordings showed that the graft-derived neurons had spontaneous action potentials, were able to fire action potentials in response to synaptic activation, and exhibited spontaneous postsynaptic currents, suggesting that these neurons were electrically active and capable of firing action potentials and of receiving synaptic inputs. Based on the firing morphology and membrane potential of the cells, they were thought to be interneurons or GABAergic neurons. The parameters of the graft-derived neurons were similar to those of the host neurons. Remarkably, the grafted NSCs-derived neurons were encapsulated by oligodendrocytes to form myelin sheaths, which contributed significantly to the impulse transport. Overall, the results indicated that graft-derived neurons derived could form mature morphological structures, have functional synaptic connections with host neurons, and integrate into the host neural circuits.

Infarct volume was reduced after the transplantation of NSCs overexpressing BDNF/Dlx2. The results of the Voetsch score, which assesses the sensorimotor ability, and the limb placement test, assessing vision, touch, and proprioception, suggested that the functional recovery from brainstem stroke was improved by transplantation of NSCs overexpressing BDNF/Dlx2.

## Conclusions

In summary, co-overexpressed BDNF and Dlx2 promotes the differentiation of NSCs into GABAergic neurons, which established synaptic connections with host neurons to reconstruct neural circuits. Our study provided evidence for the use of exogenous NSCs for transplantation in the treatment of brainstem stroke.

## Supplementary Information

**Additional file 1**: **Table S2**. Primer sequences.

**Additional file 2**: **Table S1**. Primary and secondary antibodies.

**Additional file 3**: **Fig. S1**. Endogenous NSCs in the subgranular zone of the hippocampal dentate gyrus were detected by anti-Ki67 and anti-nestin on Day 28 after brainstem infarction. *N* = 6, scale bar = 20 μm.

**Additional file 4**: **Fig. S2**. Identification of lentiviral vectors. The NSCs in the control, BDNF-OE, BDNF/Dlx2-OE, and TrkB-KD groups were transfected with Lenti-control, Lenti-BDNF, Lenti-BDNF/Dlx2, and Lenti-shTrkB, respectively. The mRNA level was measured by qRT-PCR at 3 days after transfection. Levels of BDNF and TrkB. Levels of BDNF and Dlx2. *N* = 6, *P < 0.05, vs. control group.

## Data Availability

All data generated or analyzed during this study are included in this published article.

## Abbreviations

NSCs: Neural stem cells
BDNF: Brain-derived neurotrophic factor
MCAO: Middle cerebral artery occlusion
Dlx2: Distal-less homeobox 2
TE: Echo time
TR: Repetition time
FOV: Field of view
FBS: Fetal bovine serum
ACSF: Artificial cerebrospinal fluid
PSC: Postsynaptic current
ET-1: Endothelin
SVZ: Subventricular zone
SGZ: Subgranular zone
