# Supplementary material for: GABAergic neurons differentiated from BDNF- and Dlx2-modified neural stem cells restore disrupted neural circuits in brainstem stroke
Source: Stem Cell Res Ther. 2023 Jun 26;14:170. doi: 10.1186/s13287-023-03378-5 (PMC10294474; doi:10.1186/s13287-023-03378-5)
Supplement: Supplementary file 1 — Additional file 1: Table S2. Primer sequences. [file 13287_2023_3378_MOESM1_ESM.docx]

**Table S2. Primer sequences**

| Gene | Forward (5’→ 3’) | Reverse (5’→ 3’) |
| --- | --- | --- |
| BDNF | TCATACTTCGGTTGCATGAAGG | AGACCTCTCGAACCTGCCC |
| TrKB | CTGGGGCTTATGCCTGCTG | AGGCTCAGTACACCAAATCCTA |
| Dlx2 | GTGGCTGATATGCACTCGACC | GCTGGTTGGTGTAGTAGCTGC |
| β-Actin | TGGTCGTCGACAACGGCTC | CCATGTCGTCCCAGTTGGTAAC |
