# Supplementary material for: GABAergic neurons differentiated from BDNF- and Dlx2-modified neural stem cells restore disrupted neural circuits in brainstem stroke
Source: Stem Cell Res Ther. 2023 Jun 26;14:170. doi: 10.1186/s13287-023-03378-5 (PMC10294474; doi:10.1186/s13287-023-03378-5)
Supplement: Supplementary file 2 — Additional file 2: Table S1. Primary and secondary antibodies. [file 13287_2023_3378_MOESM2_ESM.docx]

**Table S1. Primary and secondary antibodies**

| Antibodies | Species | Dilution | Source |
| --- | --- | --- | --- |
| NeuN | mouse | 1:300 | Abcam, London, UK |
| GFAP | Mouse | 1:300 | Proteintech, USA |
| Nestin | mouse | 1:200 | Abcam, London, UK |
| Ki67 | Rabbit | 1:200 | Abcam, London, UK |
| ChAT | Rabbit | 1:100 | Abcam, London, UK |
| GAD65+67 | Rabbit | 1:100 | Abcam, London, UK |
| βⅢ-Tubulin | Rabbit | 1:500 | Abcam, London, UK |
| BDNF | Rabbit | 1:500 | Abcam, London, UK |
| MAP2 | Rabbit | 1:100 | Abcam, London, UK |
| NMDAR | Rabbit | 1:100 | Signalway Antibody, USA |
| PSD95 | Rabbit | 1:200 | Cell Signaling Technology, USA |
| Synapsin | mouse | 1:100 | Santa Cruz, USA |
| MBP | Rabbit | 1:500 | Proteintech, USA |
| Alexa 488 conjugated  anti rabbit secondary  antibody | Goat | 1:500 | Jackson ImmunoResearch, USA |
| Alexa 488 conjugated  anti mouse secondary  antibody | Goat | 1:500 | Cell Signaling Technology, USA |
| Cy3 conjugated anti  rabbit secondary  antibody | Donkey | 1:500 | Jackson ImmunoResearch, USA |
| Cy3 conjugated anti  mouse secondary  antibody | Goat | 1:500 | Jackson ImmunoResearch, USA |
