# Supplementary figures and images for: GABAergic neurons differentiated from BDNF- and Dlx2-modified neural stem cells restore disrupted neural circuits in brainstem stroke
Source: Stem Cell Res Ther. 2023 Jun 26;14:170. doi: 10.1186/s13287-023-03378-5 (PMC10294474; doi:10.1186/s13287-023-03378-5)

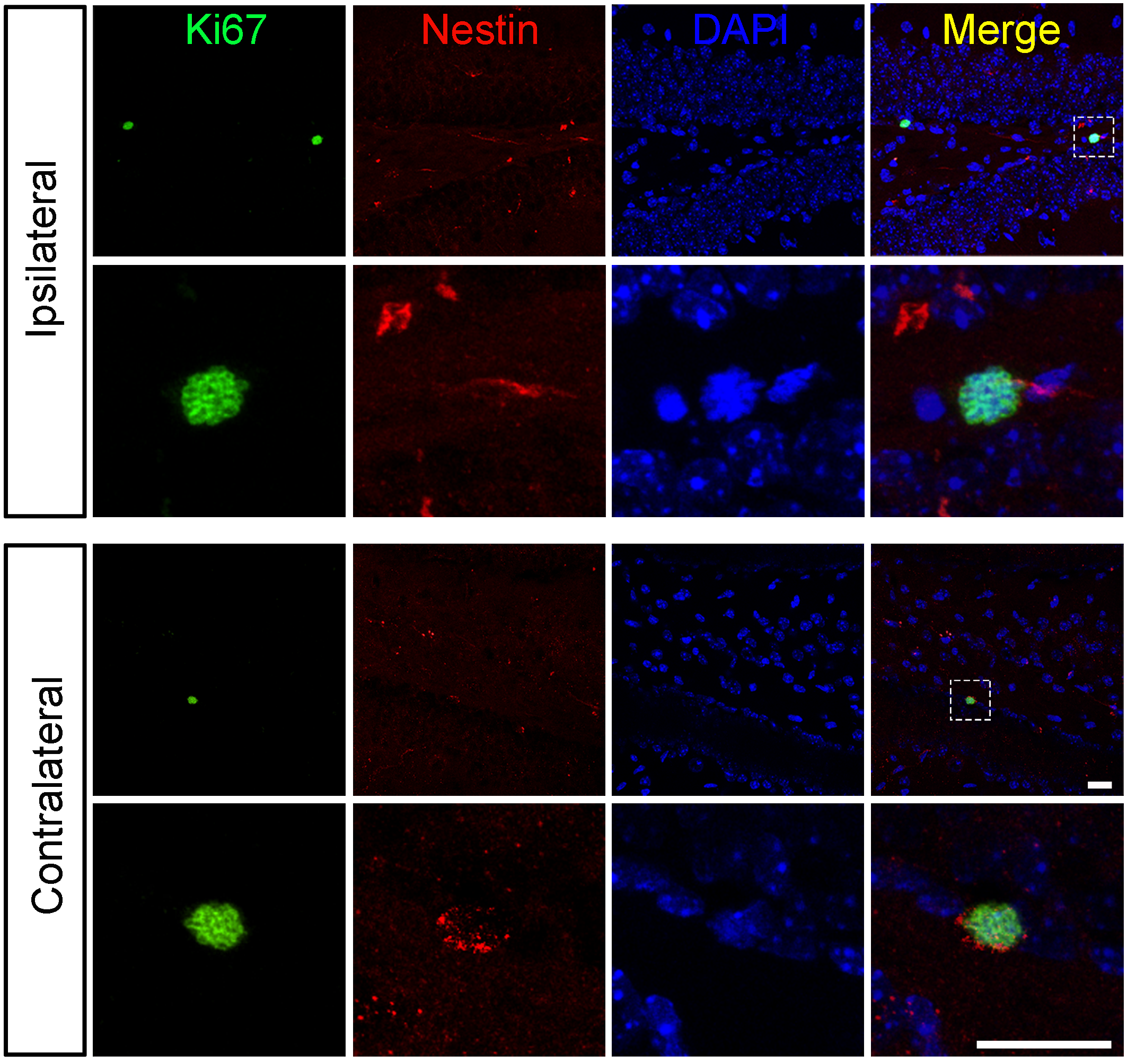

Supplement: Supplementary file 3 — Additional file 3: Fig. S1. Endogenous NSCs in the subgranular zone of the hippocampal dentate gyrus were detected by anti-Ki67 and anti-nestin on Day 28 after brainstem infarction. N = 6, scale bar = 20 μm. [file 13287_2023_3378_MOESM3_ESM.tif]

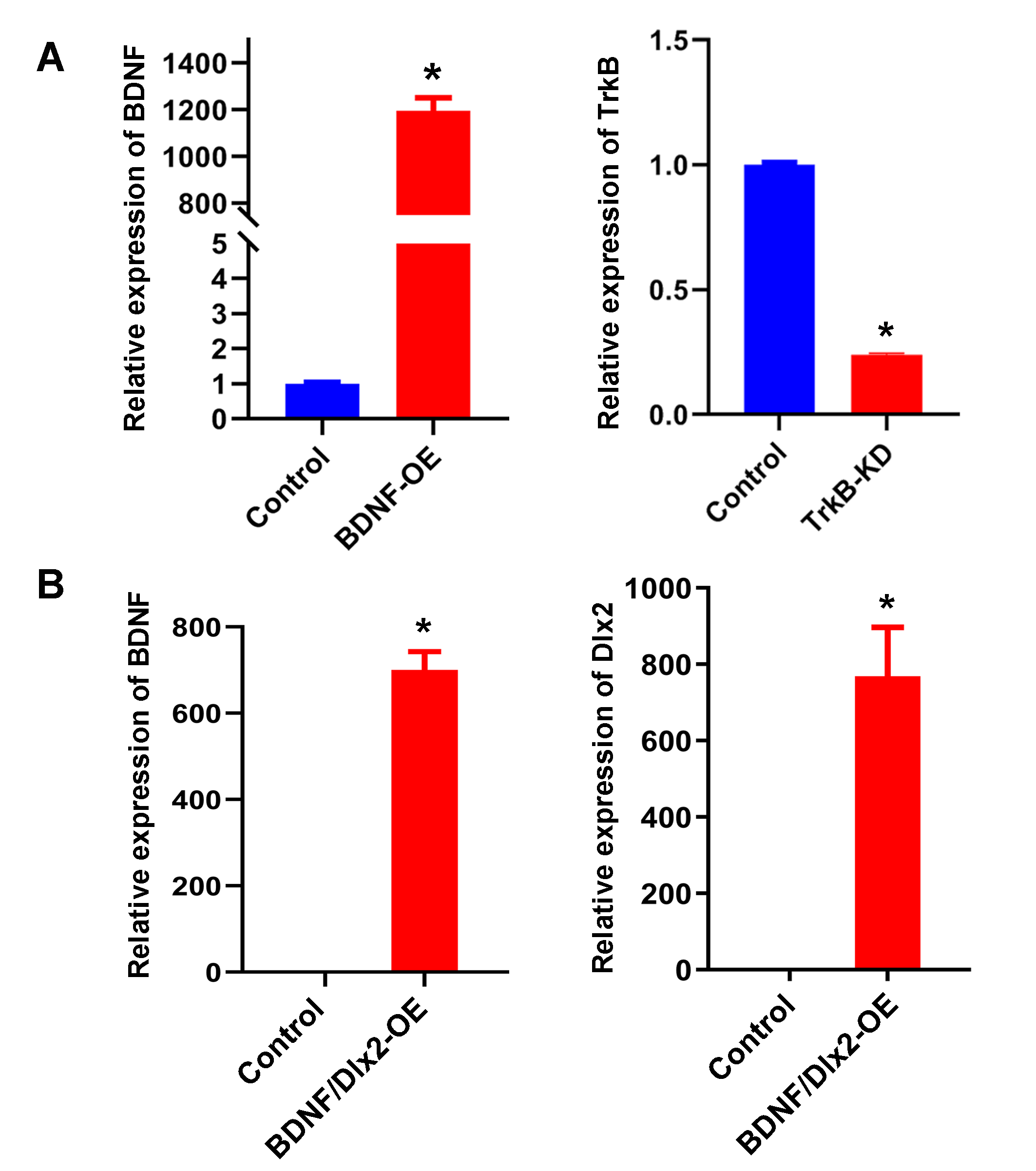

Supplement: Supplementary file 4 — Additional file 4: Fig. S2. Identification of lentiviral vectors. The NSCs in the control, BDNF-OE, BDNF/Dlx2-OE, and TrkB-KD groups were transfected with Lenti-control, Lenti-BDNF, Lenti-BDNF/Dlx2, and Lenti-shTrkB, respectively. The mRNA level was measured by qRT-PCR at 3 days after transfection. Levels of BDNF and TrkB. Levels of BDNF and Dlx2. N = 6, *P < 0.05, vs. control group. [file 13287_2023_3378_MOESM4_ESM.tif]
